# Immunotoxicity of Per- and Polyfluoroalkyl Substances: Insights into Short-Chain PFAS Exposure

**DOI:** 10.3390/toxics9050100

**Published:** 2021-05-01

**Authors:** Tracey Woodlief, Samuel Vance, Qing Hu, Jamie DeWitt

**Affiliations:** Department of Pharmacology and Toxicology, Brody School of Medicine, East Carolina University, Greenville, NC 27858, USA; vances13@students.ecu.edu (S.V.); HUQ@ecu.edu (Q.H.); DEWITTJ@ecu.edu (J.D.)

**Keywords:** PFAS: per- and polyfluoroalkyl substance, PFOA: perfluorooctanoic acid, PFOS: perfluorooctane sulfonic acid, PFMOAA: perfluoro-2-methoxyacetic acid, PFMOPrA: perfluoro-2-methoxypropanoic acid, PFMOBA: perfluoro-4-methoxybutanioc acid, NK: Natural Killer, TDAR: T cell-dependent antibody responses

## Abstract

Novel per- and polyfluoroalkyl substances (PFAS) were recently identified in drinking water sources throughout North Carolina. These include the perfluoroether acids (PFEAs) perfluoro-2-methoxyacetic acid (PFMOAA), perfluoro-2-methoxypropanoic acid (PFMOPrA), and perfluoro-4-methoxybutanioc acid (PFMOBA). Little toxicological data exist for these PFEAs. Therefore, the present study described signs of toxicity and immunotoxicity following oral exposure. Adult male and female C57BL/6 mice were exposed once/day for 30 days to PFMOAA (0, 0.00025, 0.025, or 2.5 mg/kg), PFMOPrA, or PFMOBA (0, 0.5, 5, or 50 mg/kg). A dose of 7.5 mg/kg of perfluorooctanoic acid (PFOA) was used as a positive control. Terminal body weights, and absolute liver, spleen, or thymus weights did not differ by dose for any compound; exposure to 50 mg/kg of PFMOBA increased relative liver weights in males. Changes in splenic cellularity were observed in males exposed to PFMOPrA and decreased numbers of B and natural killer (NK) cells were observed in males and females exposed to PFMOBA. Exposure did not alter NK cell cytotoxicity or T cell-dependent antibody responses at doses administered. Our results indicate that these “understudied” PFAS have toxicological potential but require additional investigation across endpoints and species, including humans, to understand health effects via drinking water exposure.

## 1. Introduction

Per- and polyfluoroalkyl substances (PFAS) are fluorinated synthetic organic substances used in a wide variety of industrial and consumer processes and products and are now widespread environmental contaminants. The vast majority of PFAS are extremely persistent; the strength of the carbon–fluorine bond confers thermal and chemical stability as well as resistance to metabolic breakdown [1].Current estimates by the U.S. Environmental Protection Agency (U.S. EPA) number PFAS at over 9000 individual chemicals and counting, none of which are currently regulated at the federal level as environmental contaminants within the U.S. [2]. The Centers for Disease Control and Prevention (CDC) monitors for 14 individual PFAS in serum and urine collected from a cross-section of the U.S. general population through the National Health and Nutrition Examination Survey [3]. This biomonitoring program has indicated that at least 98% of those sampled had detectable concentrations of PFAS in serum or urine, indicating that PFAS contamination is also widespread among humans [4].

The bulk of epidemiological and toxicological knowledge concerns a subset of PFAS known as perfluoroalkyl acids (PFAAs), which includes perfluorooctanoic acid (PFOA) and perfluorooctane sulfonic acid (PFOS). A host of adverse health outcomes have been identified in people exposed to PFAAs because they work with PFAAs, live in areas that have high levels of PFAAs in the environment, or are even exposed from everyday activities. These outcomes include effects on the liver, the cardiovascular, endocrine, immune, and reproductive systems, and on development [5]. Some populations have also seen increases in kidney and testicular cancer associated with PFOA exposure [5]. These adverse health effects also have been observed in experimental animals exposed to individual PFAAs through food or water, which are supportive of these findings of adverse health effects in humans. While “long-chain” PFAAs have been phased out of production for most processes in the U.S. due to their persistence, ability to bioaccumulate, and toxicity (i.e., “PBT” properties), they still remain in the environment and living organisms will still be exposed into the foreseeable future. Monitoring of various U.S. drinking water sources determined that levels of two long-chain PFAAs, PFOA and PFOS, exceed the U.S. EPA lifetime health advisory level (70 ng/L) for more than six million Americans [6]. Short-chain PFAS designed to take the place of long-chain PFAS in products and processes have also been detected in the environment, raising concerns about their PBT properties.

One group of PFAS that have raised human and environmental health concerns, especially in the state of North Carolina (NC), are known as perfluoroalkyl ether acids (PFEA). These include a compound known as hexafluoropropylene oxide-dimer acid (HFPO-DA or the trade name “GenX”), which is a replacement for PFOA as a processing aid in the production of fluoropolymers [7]. GenX and related compounds were measured in the Cape Fear River water as well as finished drinking water of more than 200,000 NC residents [7] and stimulated not only community concern and activism but a financial commitment by the NC General Assembly for PFAS research. This was accomplished through an infusion of state dollars to the NC Policy Collaboratory, an entity established to facilitate the dissemination of policy and research expertise at state institutions of higher education for practical use by state and local governments [8]. The NC Policy Collaboratory established the “PFAS Testing Network”, a collection of scientists from seven different universities in NC, to investigate levels and types PFAS in water and air, to explore treatment technologies, and to better understand their sources, fates, and health effects [9]. The studies described in this manuscript are part of the efforts of the PFAS Testing Network.

In this study, we focused on three PFEAs: perfluoro-2-methoxyacetic acid (PFMOAA), 2,2,3,3-(trifluoromethoxy) propionic acid (PFMOPrA), and perfluoro-4-methoxybutanioc acid (PFMOBA). Each of these PFEAs has been detected in the Cape Fear River and this group represents carbon chain lengths of three, four, and five, respectively. PFMOAA, PFMOPrA, and PFMOBA are thought to be byproducts of the GenX manufacturing process and were not part of any allowable discharges covered under National Pollutant Discharge Elimination System (NPDES) permits by the manufacturing facility that discharges into the Cape Fear River [10].

Due to their environmental persistence, ability to bioaccumulate, and a growing body of toxicological evidence associated with their exposure, PFOA and PFOS have been mostly phased out of production and use in the U.S. However, phase-outs of PFOA, PFOS, and other long-chain PFAAs have led to a rise in the production of alternatives, touted as having more favorable environmental and toxicological properties [11,12,13,14,15]. Additionally, in 2016, the U.S. National Toxicology Program (NTP) published a systematic review of PFOA and PFOS and classified them as presumed to be immune hazards to humans [16]. Therefore, we also wanted to determine whether PFMOAA, PFMOPrA and PFMOBA, newly discovered in the Cape Fear River, would produce signs of immunotoxicity as defined by harmonized testing guidelines for immunotoxicity [17]. To our knowledge, little to no toxicological data exist for these compounds in the peer-reviewed literature.

## 2. Materials and Methods

### 2.1. Animals

All experimental animal handling and dosing was carried out in accordance with procedures approved by East Carolina University’s Institutional Animal Care and Use Committee (IACUC); all animals were treated humanely and with regard to alleviation of suffering. Male (75) and female (75) C57BL/6 mice, 6–8 weeks old (Charles River Laboratories), were ordered and delivered in three separate batches; experiments for each PFAS were independently conducted. For each PFAS, mice were randomly assigned to groups of 3/cage. The C57BL/6 mouse strain was utilized for consistency with previously published findings [18,19,20]. Animals were weighed upon delivery and weights by cage were evaluated by analysis of variance (ANOVA). Animals within cages were adjusted, if needed, to ensure that weights did not differ statistically at the start of the study. Two cages of three animals each (six animals) per sex were randomly assigned to dose groups. Animals were housed with access to food and water ad libitum, with a 12:12 h light–dark cycle at 22 ± 3 °C and 50 ± 20% humidity. Animals were acclimated for at least 5 days before dosing began.

### 2.2. Dosing

Dosing solutions were prepared fresh weekly in sterile water with 0.5% Tween-20 at the following concentrations (PFMOAA: 0.000025 mg/mL, 0.0025 mg/mL, 0.25 mg/mL; PFMOPrA and PFMOBA: 0.05 mg/mL; 0.5 mg/mL; 5.0 mg/mL). Vehicle control mice received sterile water only (+0.5% Tween-20). PFMOAA was gifted by Dr. Ralph Mead from the University of North Carolina-Wilmington. PFMOPrA and PFMOBA were purchased from SynQuest. PFOA was purchased from Sigma. Dosing was performed daily for 30 consecutive days based on individual daily body weights. Dosing solutions were administered at 0.1 mL/10 g of body weight, resulting in PFMOAA doses of 0, 0.00025, 0.025, and 2.5 mg/kg, and in PFMOPrA and PFMOBA doses of 0, 0.5, 5.0, and 50.0 mg/kg. A dose of 7.5 mg/kg PFOA consisting of six animals/sex was used as a positive control for well-known markers of toxicity/immunotoxicity. These animals were dosed with another batch of animals exposed to a different PFAS, but all of the experimental conditions were consistent with this set of experiments. The 7.5 mg/kg dose of PFOA has been demonstrated to produce immunotoxicity in the absence of overt systemic toxicity [18]. Data from animals exposed to PFOA included body weight, liver weight, lymphoid organ weight, peroxisome proliferation, and the TDAR as these are known to be sensitive to PFOA exposure. Data for other endpoints (immunophenotype and NK cell cytotoxicity) following PFOA exposure were not used as comparators to the results for the three PFEAs evaluated in this manuscript as these endpoints are not as sensitive to PFOA following adult exposures. Dosing concentrations for the PFEAs were based on reports of concentrations of these compounds measured in finished water from the Cape Fear River [10] as well as anticipated immunotoxicity associated with doses of PFOA. Those administering dosing solutions were not blinded to group identification to prevent cross-contamination among groups. It should be noted that during the first week of dosing male animals with PFMOPrA, the doses were increased by a factor of two due to miscommunication with the vendor about the stock concentration of PFMOPrA. Animals received the appropriate doses in the subsequent three weeks and female animals received the appropriate doses during all weeks of exposure. While no signs of systemic toxicity were observed in the males during the first week of dosing at the higher administered concentration, we cannot discount that this may have impacted results. On the 26th day after the initial dose, all mice in all dose groups were immunized with 4 × 10^7^ sheep red blood cells (SRBCs) in 0.2 mL saline via tail vein injections to ensure that SRBC-specific IgM was at peak serum concentrations five days later, at euthanasia (DeWitt et al., 2008).

### 2.3. Body and Organ Weights

Body weights were recorded daily and were used, along with physical observations such as activity, posture, and body condition, over the course of the study to determine potential overt toxicological responses. Animals that experienced ≥20% weight loss during the exposure period were removed from the study; when this occurred, animals were humanely euthanized and necropsied. Immediately following euthanasia, spleen, thymus, brain, liver, heart, lungs, and kidneys were removed and weighed. All organs except for spleen, thymus, and heart were frozen at −80 °C for future analysis. Fresh spleen and thymus were placed in 6-well plates filled with RPMI 1640-medium (supplemented with 1% fetal bovine serum (FBS) and kept on ice for immunophenotyping and natural killer (NK) cell activity assays. Hearts were not retained.

### 2.4. Serum Preparation

Following anesthesia via isoflurane, blood was collected by neck vein transection into microcentrifuge tubes with a clot activator, allowed to sit for ~30 min, then was centrifuged at 14,000 rpm for 7 min at 4 °C. Serum separated from the clot was collected and then frozen at −80 °C until further analysis. Samples of serum were pooled per cage and sent to Enthalpy Labs (Wilmington, NC, USA) for analysis of PFMOAA, PFMOPrA or PFMOBA concentrations using the isotope dilution method using Waters Acquity UPLC equipped with Xevo TZ MS (LC/MS/MS). Remaining serum was retained for evaluation of SRBC-specific antibody responses.

### 2.5. Immunophenotyping

Immunophenotyping was performed as previously described [21]. In brief, individual spleens and thymuses were processed into single-cell suspensions and passed through 70 µm filters. Cell suspensions were counted on a Nexcelon Bioscience Cellometer Auto 2000 cell counter (Nexcelom Bioscience, Lawrence, MA, USA) to determine total cell count and viability, then adjusted to 2 × 10^7^ cells/mL. Spleen cell suspensions were divided into two aliquots, one for T cells and one for B and natural killer (NK) cells, for counting by flow cytometry. Monoclonal antibodies (eBioscience, San Diego, CA, USA) coupled to fluorochrome specific markers were used as follows: APC anti-mouse CD3, FITC anti-mouse CD4, and PE anti-mouse CD8a (spleen and thymus samples); FITC anti-mouse CD45R and PE anti-mouse NK1.1 (spleen only). All experimental replicates also included unstained cells (negative control) and single-color (positive control) to determine color compensation. Flow cytometric analysis was performed using an BD LSRII flow cytometer (BD sciences, Indianapolis, IN, USA) with 10,000 events collected per sample. Dead cells and debris were excluded from analysis by using forward scatter and 90° light scatter to establish a gate around viable lymphocyte populations. Non-stained cells, singe-color controls, and fluorescence minus one (FMO) controls were used to distinguish the negative populations from the positive populations for B cells, NK cells, and T cells. Cells were gated based on CD3 expression for subsequent analysis of CD4/CD8 T-subpopulations, but not B and NK cell subpopulations. The total number of each cell type was determined from the specific organ cellularity.

### 2.6. T Cell-Dependent Antibody Response (TDAR)

TDAR was assessed as previously described but with some modifications [22]. Mouse anti-SRBC IgM was evaluated in serum using pre-coated 96-well enzyme-linked immunosorbent assay (ELISA) plates (Life Diagnostics, West Chester, PA, USA). Serum samples were diluted 25, 50 or 75-fold and were added to plates, along with anti-SRBC IgM standards in duplicate. Wells containing just the diluent were included as blanks. Plates were incubated at room temperature for 45 min on an orbital shaker (75 rpm). Wells were then washed (5×) with 1× wash solution, tapped dry and 100 µL enzyme conjugate was added per well. Plates were then incubated at room temperature for 45 min on an orbital shaker (75 rpm), washed (5×) with 1× wash buffer and tapped dry. A volume of 100 µL TMB reagent was added per well and incubated for 20 min on an orbital shaker (75 rpm), followed by addition of 100 µL stop solution to each well. A microplate reader was used to measure absorption at 450 nm. Results were calculated by computing a standard curve based on absorbance of the standards and fitting each sample onto this curve to solve for the concentration of anti-SRBC IgM (units/mL).

### 2.7. Natural Killer (NK) Cell Assay

Preparation of target cells: YAK-1 cells were re-suspended at a concentration of 2 × 10^6^ cells/15 mL in RPMI^CELL^ (500 mL RPMI 1640 + 50 mL FBS + 5 mL Pen-Strep + 5 mL L-Glutamine) approximately 5 days before the NK cell assay day. Cells were spilt every 2 days and then again one day prior to the assay day. Three YAK-1 controls were prepared as follows: 1—unstained control (no MitoTracker green; MTgreen), 2—positive control (total lysis by triton-X), 3—negative control (spontaneous lysis no added effector cells). All YAK-1 cells except for the unstained controls were incubated in 300 nM MTgreen for 20 min at 37 °C, centrifuged at 14,000 rpm for 5 min, and resuspended in RPMI^ASSAY^ (RPMI 1640 + 10% FBS) until effector cells were ready. Preparation of effector cells: splenic lymphocytes were isolated and prepared as described under “Immunophenotyping” and were diluted to 1 × 10^6^/mL, centrifuged at 14,000 rpm for 5 min, and resuspended in 500 µL of RPMI^ASSAY^. Target cells (500 µL) were added to each tube of effector cells (Effector cell: Target cell (E:T)), and incubated for 3 h at 37 °C. Three E:T ratios were used in this study, 5:1, 10:1 and 30:1. Post incubation, 15 µL of propidium iodine solution was added and incubated for 5–15 min prior to flow analysis. Percent specific lysis was calculated as follows: ((sample dead cells-control spontaneous lysis))/(sample live cells + (sample dead cells-control spontaneous lysis)) × 100. NK cell activity was not assessed for animals exposed to PFMOAA. NK cell activity for the female cohort in response to PFMOBA is only represented by one E:T ratio and in response to PFMOPrA is only represented by two E:T ratios due to limited YAK cells.

### 2.8. Liver Peroxisome Proliferation

Liver peroxisome proliferation was measured as previously described [13,21,23]. In brief, peroxisome proliferation was measured indirectly by acyl CoA oxidase activity in liver homogenates, utilizing the H_2_O_2_-dependent oxidation of 0.05 mM leuco-DCF to DCF. Total protein concentration of the supernatant was determined by Braford protein assay. Peroxisome proliferation data were expressed as percent change from the control (0 mg/kg).

### 2.9. Statistical Analysis

Statistical analyses were performed using the Statistical Analysis System (SAS). Dosing body weights were collected daily and analyzed by two-way analysis of variance (ANOVA) with dose as the between-subjects variable and day as the within-subjects variable. Remaining data were analyzed within each sex by one-way ANOVA by dose. If the F-statistic was statistically significant for the overall model by dose, pairwise post hoc t-tests were made with a Tukey’s adjustment for the familywise error rate. Statistical significance was determined with an alpha of 0.05.

## 3. Results

### 3.1. Serum PFMOAA, PFMOPrA or PFMOBA Concentrations

PFMOAA or PFMOBA levels were not observed above the level of detection (LOD) in serum of male or female animals collected one day after the 30 day daily oral exposures. In male animals exposed to PFMOPrA, serum concentrations of PFMOPrA increased in a dose-responsive manner from the 0 mg/kg group (<LOD), to 48 ng/mL (0.5 mg/kg group), 366 ng/mL (5.0 mg/kg group) and 872 ng/mL (50 mg/kg group). In female animals exposed to PFMOPrA, serum concentrations in both the 0 and 0.5 mg/kg group were below the LOD whereas the 5 and 50 mg/kg dose groups had detectable concentrations of 4.13 and 28.40 ng/mL, respectively (Supplemental Table S1). The LODs were as follows: (PFMOAA 0.5 ng/mL, PFMOPrA 1.2 ng/mL, PFMOBA 7.4 ng/mL).

### 3.2. Body and Liver Weights

Body weights remained consistent among doses over the dosing period in both male and female animals for all three PFEAs evaluated as well as for animals given PFOA. Terminal body weights did not differ for animals given the PFEAs nor did they differ for male animals given PFOA (Supplemental Tables S2 and S3). Terminal body weights of female animals exposed to PFOA were statistically (*p* < 0.05) decreased by 19.3%, but this was largely driven by one animal that lost weight late in the dosing period (Supplemental Table S3). Relative liver weights of the highest-dose groups of male animals increased by 4% and 28%, respectively compared to the control groups in response to PFMOAA and PFMOPrA, but these increases were not statistically significant. Relative liver weights increased by ~15% in male animals exposed to 50 mg/kg of PFMOBA (*p* < 0.05). compared to the control group

Although not statistically significant, relative liver weights of the highest-dose groups of female animals increased by 7% and 8% compared to the control groups in response to PFMOAA and PFMOBA, respectively. Changes in relative liver weights were not observed in female animals exposed to PFMOPrA (Supplemental Table S3). In the PFOA-positive control group, relative liver weights statistically (*p* < 0.05) increased in both the male and females compared to control (male 138%; female 210%; Supplemental Tables S2 and S3).

No overt signs of toxicity, such as changes in body weight, activity, posture, or body condition were observed in any of the animals in response to PFMOAA, PFMOPrA, PFMOBA, or PFOA. A total of eight animals were removed from the three PFEA studies due to body weight loss and one animal was removed from the PFOA study; upon necropsy, these animals had gavage-related injuries to the esophagus/thoracic cavity that did not appear to be related to administered substance toxicity.

### 3.3. Lymphoid Organ Weights and Immune Organ Cellularity.

No statistical differences in thymus or spleen weights were observed in any of the PFEA dose groups compared to control groups (Supplemental Tables S2 and S3). The PFOA-positive control group had a 40% reduction (*p* < 0.05) in the relative spleen weights in female but not male animals.

No statistical differences were detected in thymus cellularity in male or female animals given PFMOAA, PFMOPrA or PFMOBA compared to control (Supplemental Tables S4 and S5) and no statistical differences in splenic cellularity were detected in male animals given PFMOAA, PFMOPrA, or PFMOBA compared to controls (Supplemental Table S6). Female animals given 0.5 and 50 mg/kg of PFMOPrA had an 11.2% and 23.1% decrease in splenic cellularity, respectively and female animals given 5 mg/kg of PFMOPrA had a 19.4% increase in splenic cellularity relative to controls (*p* < 0.05; Supplemental Table S7).

### 3.4. Immunophenotyping Thymus

No statistical differences were detected in thymus T cell subpopulations in male or female animals given PFMOAA, PFMOPrA or PFMOBA compared to control group (Supplemental Tables S4 and S5).

### 3.5. Immunophenotyping Spleen

No statistical differences were detected in any of the spleen cell populations measured in male or female animals given PFMOAA or PFMOPrA (Supplemental Table S8).

In male and female animals exposed to PFMOBA, several statistical (*p* < 0.05) changes were observed in the numbers of B and NK cell subpopulations within the spleen (Supplemental Table S8). In female animals exposed to 50 mg/kg of PFMOBA, numbers of B cells decreased by 47.9% and numbers of NK cells decreased by 54.2%. In male animals, all doses of PFMOBA increased numbers of B cells and NK cells, on average by 87.3% and 94.4%, respectively (Supplemental Table S8).

### 3.6. Peroxisome Proliferation (ACOX-1 Activity)

**Figure 1:**
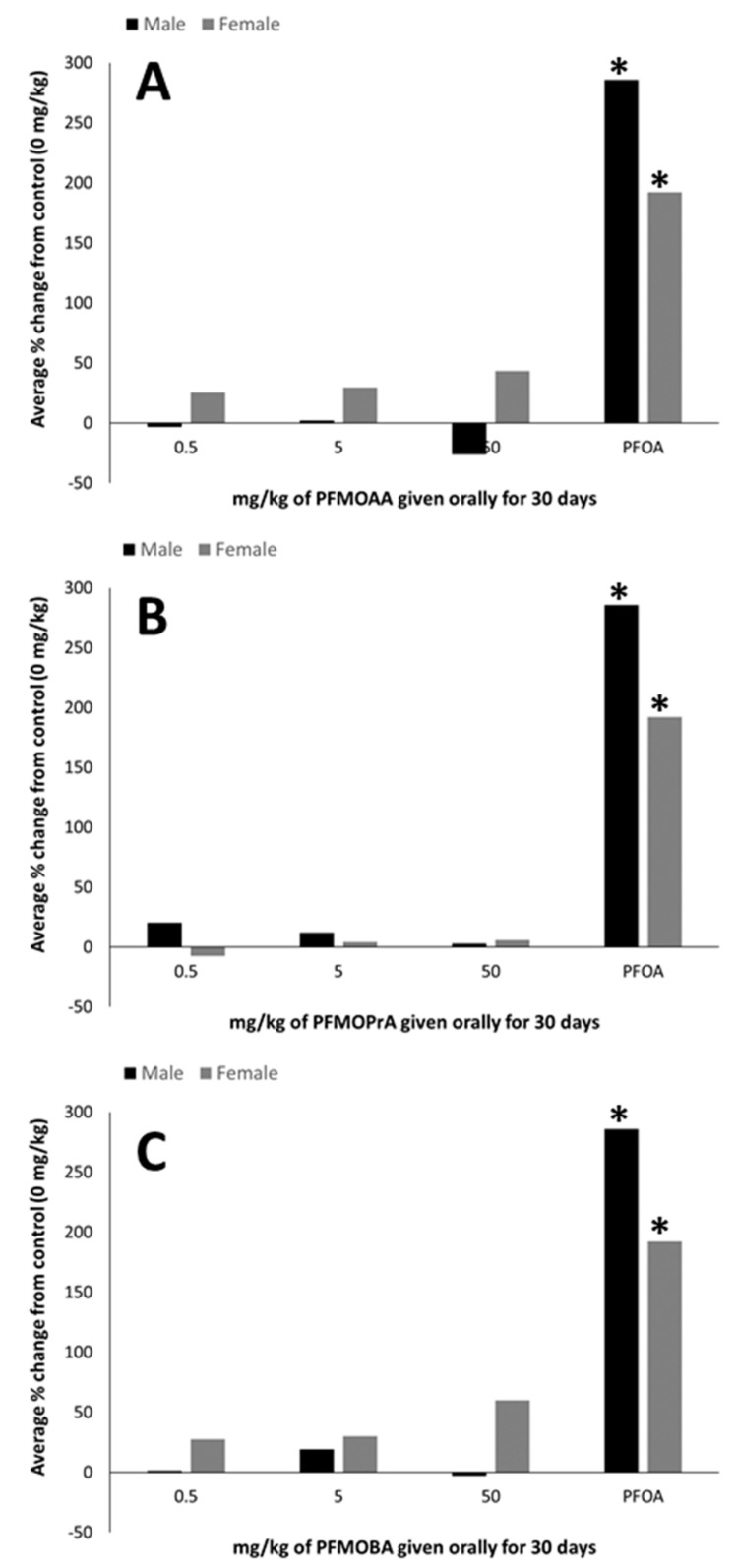
Hepatic peroxisome proliferation (percent change from 0 mg/kg control) of male and female C57BL/6 mice orally exposed to (**A**): PFMOAA, (**B**): PFMOPrA, or (**C**): PFMOBA for 30 days. Acyl-CoA oxidase activity was measured in livers that had been collected from animals one day after exposure ended. *n* = 4–6/dose for PFMOAA, PFMOPrA, PFMOBA, and PFOA-positive control (note that the PFOA-positive control was included from animals evaluated in a separate PFAS study). No error bars are present due to how the data were calculated. Abbreviations: perfluoro-2-methoxyacetic acid (PFMOAA), perfluoro-2-methoxypropanoic acid (PFMOPrA), perfluoro-4-methoxybutanioc acid (PFMOBA), and perfluorooctanoic acid (PFOA). * *p* < 0.05 from same-sex control group.

In male and female animals given PFMOAA, PFMOPrA, or PFMOBA, no statistical differences were detected in the percent change in ACOX-1 activity from control groups (Figure 1). In both the male and female positive control groups, exposure to PFOA resulted in an approximate 200% increase in ACOX-1 activity compared to controls (0 mg/kg).

### 3.7. NK Cell Cytotoxicity

**Figure 2:**
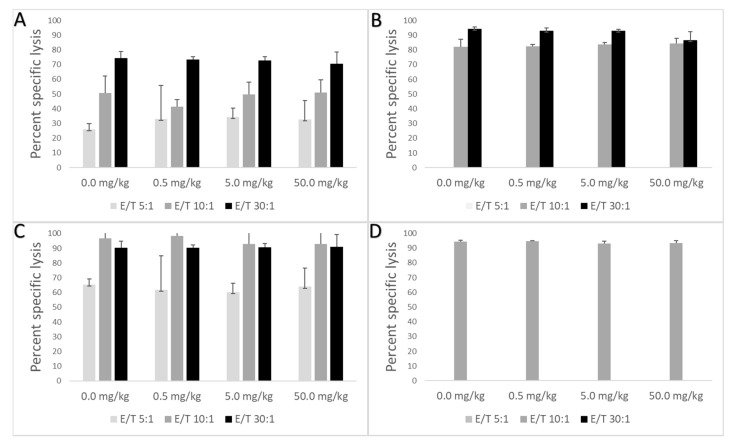
Mean percent specific lysis (± standard deviation) in male and female C57BL/6 mice orally exposed to PFMOPrA or PFMOBA for 30 days. Cells were analyzed by flow cytometry one day after dosing ended. (**A**): Male PFMOPrA; (**B**): female PFMOPrA; (**C**): male PFMOBA; (**D**): female PFMOBA. Percent specific lysis was not measured in response to PFMOAA or PFOA and an insufficient number of YAK-1 cells were available for all three E:T ratios for female animals exposed to PFMOPrA or PFMOBA. *n* = 4–6/dose. Abbreviations: E (effector cells); T (target cells); perfluoro-2-methoxyacetic acid (PFMOAA), perfluoro-2-methoxypropanoic acid (PFMOPrA), perfluoro-4-methoxybutanioc acid (PFMOBA).

No statistical differences were detected in NK cell activity in male or female animals given PFMOPrA or PFMOBA compared to control groups (Figure 2).

### 3.8. TDAR

**Figure 3:**
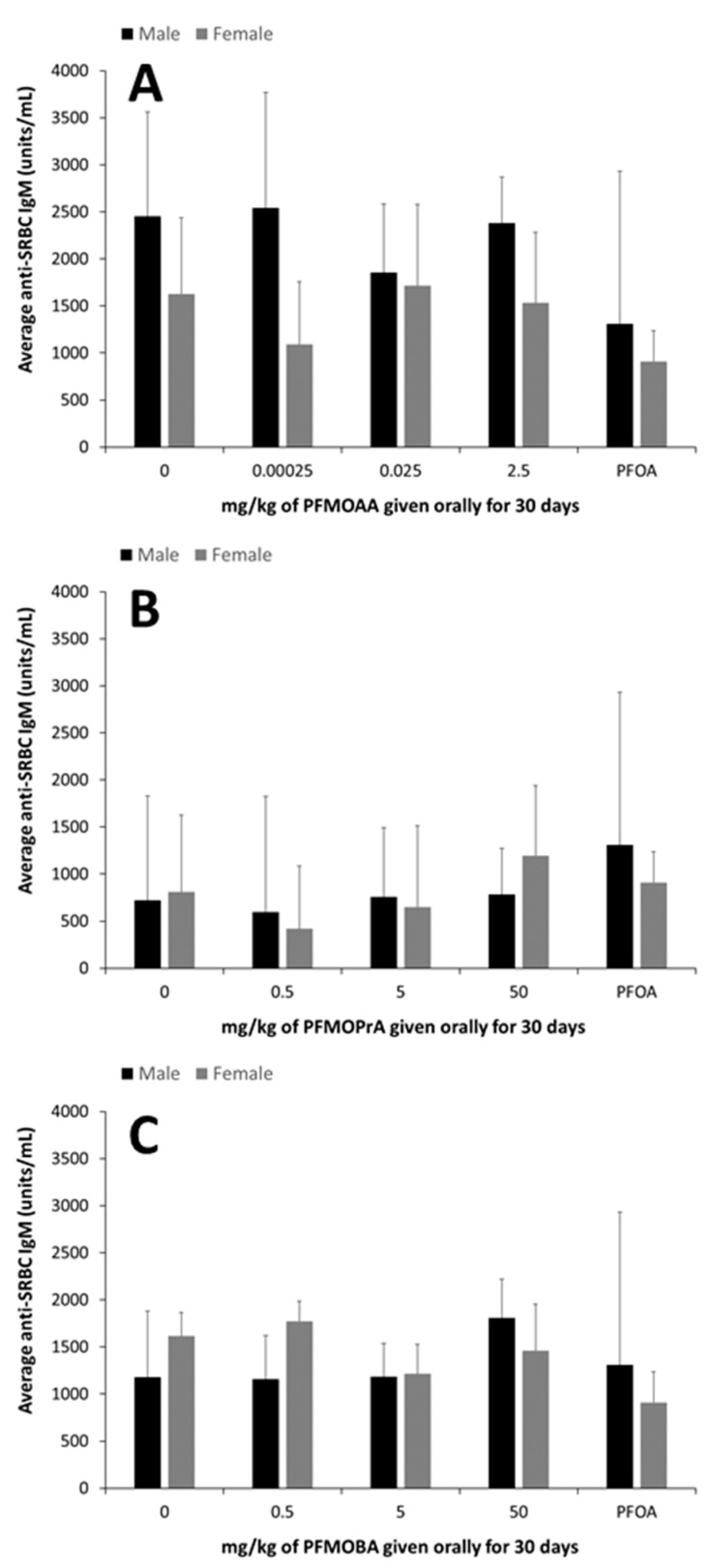
Mean anti-SRBC IgM (units/mL) (± standard deviation) in male and female C57BL/6 mice orally exposed to (**A**): PFMOAA, (**B**): PFMOPrA, or (**C**): PFMOBA for 30 days. Serum was analyzed one day after dosing ended and five days after immunizations. No statistical differences in the TDAR were detected in male or female animals given PFMOAA, PFMOPrA, or PFMOBA (Figure 3). *n* = 4–6/dose for PFMOAA, PFMOPrA, PFMOBA, and 2–3/dose for PFOA-positive control (note that the PFOA-positive control was included from animals evaluated in a separate PFAS study). Abbreviations: sheep red blood cell (SRBC); T cell-dependent antibody response (TDAR); perfluoro-2-methoxyacetic acid (PFMOAA), perfluoro-2-methoxypropanoic acid (PFMOPrA), perfluoro-4-methoxybutanioc acid (PFMOBA), and perfluorooctanoic acid (PFOA).

No statistical differences were detected in the TDAR in male or female animals given PFMOAA, PFMOPrA, PFMOBA, or PFOA compared to control groups (Figure 3).

## 4. Discussion

To our knowledge, this is the first report of effects of PFEAs on general toxicity and immunotoxicity endpoints (in-life observations, organ weights, spleen and thymus immune-cell populations, NK cell activity, SRBC-specific IgM production) following oral exposure in a rodent model. In addition, we measured peroxisome proliferation, a well-accepted biomarker of exposure to legacy or long-chain PFAAs. Overall, our goal was to determine whether these PFEAs were capable of producing immunotoxicological outcomes similar to those produced by exposure to PFOA. We choose these endpoints as they been shown to be altered in response to PFOA or PFOS exposure and are robust markers of immunotoxicity and predictive of human immunotoxicological health risks [13,22,24,25]. Little to no toxicological data exist in the published literature for PFEAs, even though the extent of contamination within various water sources and in human blood samples are starting to be reported [24].

Human exposure to PFAS is a concern that prompted local, state, and federal agencies and entities to begin to investigate environmental levels and adverse effects of PFAS on living organisms. To date, more than 4000 PFAS have been manufactured, with many more being detected within environmental samples [25]; it has been estimated that almost 10,000 individual PFAS exist. Levels of legacy PFAS, though decreasing in human serum, have been demonstrated to be stable or increasing in water and seafood samples [25]. Relative concentrations of other PFAS have been difficult to discern as not all PFAS have been identified and/or have analytical standards available. Addressing this data gap concerning the PFAS exposome is vital for determining the potential adverse effects of exposure to PFAS and/or PFAS mixtures and for prioritizing action plans to minimize health risks.

Associations between PFAA exposure and various disease states in exposed human populations have been uncovered in myriad epidemiological studies, indicating that PFAAs can induce cancer and reduce immune response. However, the results are mixed with regard to the types of immunological changes and degree of vaccine suppression [15,26,27,28,29,30,31,32]. The findings of these epidemiological studies are strengthened when combined with toxicological evidence from experimental animals. The data for immunotoxicity are especially compelling given the concordance between epidemiological studies demonstrating decreased responses to vaccines in populations environmentally exposed to PFAS and decreases in the TDAR in animals experimentally exposed to individual PFAS [18,33,34,35,36].

In previous studies of mice orally exposed to PFOA for 15 days, body weights and lymphoid organ weights were decreased by doses of 15 and 30 mg/kg compared to controls [18,24]. However, these doses were overtly toxic. In this study, the findings for the studied PFEAs were compared to a PFOA exposure of 7.5 mg/kg, which has been demonstrated to be immunotoxic independent of overt toxicity when given for only 15 days [24]. This PFOA dose was therefore chosen as a comparator that was likely to induce immunotoxicity independent of overt/systemic toxicity. We did not observe signs of overt toxicity with this dose of PFOA in terms of in-life observations. While terminal body weights of female animals were statistically decreased relative the control group, this was largely driven by one animal that lost weight in the last two days of the dosing period. Body weight was not altered by exposure to PFMOAA, PFMOPrA, or PFMOBA at the doses administered. This indicates that at these doses, these PFEAs were not overtly toxic. Effects on systemic toxicity cannot be discounted within the male cohort exposed to PFMOPrA as their does where increased by a factor of two within the first week of dosing, but as their body weights did not decrease during the dosing period, it is unlikely.

Increases in liver weight and liver peroxisome proliferation, as measured indirectly by ACOX-1 activity, are well-known biomarkers of PFOA exposure [20,37]. Peroxisome proliferation within the liver has also been demonstrated to occur before certain immunotoxic effects of PFOA, such as atrophy of the spleen and thymus [19]. In this study, the PFOA-positive control group induced increases in both relative liver weight (~210% in females; 138% in males) and peroxisome proliferation (~200% in females and males), as expected. Relative liver weights and peroxisome proliferation did not differ statistically in response to PFMOAA or PFMOPrA at the doses administered within male or female animals. In male animals exposed to PFMOBA, relative liver weights increased by approximately 15% in the high dose group relative to control weights, while there were no differences in the female animals. As these PFEAs have three, four, and five carbons relative to the eight carbons of PFOA, the lack of robust and statistically significant ACOX-1 activity suggests that these shorter carbon chain PFEAs are not as potent at inducing this response, at least at the doses administered in this study.

The immune system is well known to be sensitive to the effects of environmental exposures to PFOA and PFOS [13,22,24,37,38,39]. Evaluation of immunotoxic potential is one key endpoint that can be used to evaluate the impact of PFAS on human health and disease potential. The TDAR is a highly sensitive functional assay for evaluating immunotoxic potential and has broad applicability and translatability to understanding human health risks [13,22,24,25]. In mice exposed to lower doses of PFOA for up to 15 days, suppression of the immune response has been linked to changes in cellular function, rather than direct lymphotoxicity, as reductions in the TDAR and the T cell-independent antibody responses have been observed in the absence of changes in immune populations within the spleen [24]. In this study, the administered dose of 7.5 mg of PFOA/kg did not suppress the TDAR, but this was likely was due to the relatively small sample size of PFOA-exposed animals used for this assay combined with high variability in the assay. It is also possible that this longer PFOA exposure duration led to differences in internal dynamics with respect to antibody production. This suggests that additional studies with PFOA and even other long-chain PFAS are warranted to better understand the impacts of exposure duration on the TDAR and potentially other immune functional endpoints. The administered doses of PFMOAA, PFMOPrA and PFMOBA did not modulate the immune system as measured by changes in lymphoid organ weights, NK cell activity, or the TDAR. However, some shifts in spleen cellularity were observed following exposure to PFMOPrA in female animals and in both male and female animals, shifts in B and NK cell populations were observed following PFMOBA exposures Without concomitant changes in immune functional changes and without clear dose-responsivity of these changes in immunophenotype, the meaning of these shifts are challenging to interpret. Regardless, these changes in immunophenotype suggest that these PFEAs can induce immunological shifts in response to exposure that could perturb normal physiology and change with shorter or longer exposure durations and/or changes in exposure concentrations. Previous studies have demonstrated sporadic responses within specific immune cell populations dependent on the PFAS administered, exposure concentration, and duration of exposure [13,18,22,40].

Further studies are needed to determine the potential health effects of these novel PFAS within various species to delineate exposure measures for hazard and risk estimation and to uncover their impact on the environment, as well as human health and disease.

## 5. Conclusions

Doses of PFMOAA (0.00025, 0.025 and 2.5 mg/kg), PFMOPrA (0.5, 5.0 and 50 mg/kg), or PFMOBA (0.5, 5.0 and 50 mg/kg) administered orally for 30 days to C57BL/6 male and female mice did not alter body, liver, or lymphoid organ weights. In addition, at the doses administered, PFMOAA, PFMOPrA, and PFMOBA did not alter peroxisomal enzyme activity or immune cell function as measured by NK cell activity or the TDAR. Statistically significant shifts in immune cell populations, however, were detected. These data suggest that these PFEAs, at the doses administered, have toxicological potential, and require additional studies to determine their health effects via drinking water exposure.

## Supplementary Materials

The following are available online at https://www.mdpi.com/article/10.3390/toxics9050100/s1, Table S1: Mean (±SD) serum PFMOAA, PFMOPrA or PFMOBA concentrations in male and female C57BL/6 mice (*n* = 6/dose) orally exposed to PFMOAA, PFMOPrA or PMFOBA for 30 days, Table S2: Mean (±SD) body and relative organ weights of male C57/BL6 mice (*n* = 6) orally exposed to PFMOAA, PFMOPrA or PFMOAA for 30 days, Table S3: Mean (±SD) body and relative organ weights of female C57/BL6 mice (*n* = 6) orally exposed to PFMOAA, PFMOPrA or PFMOAA for 30 days, Table S4: Mean (±SD) number of thymic lymphocytes, adjusted to the total number of cells in the thymus (cellularity) of male C57BL/6 mice (*n* = 6) orally exposed to PFMOAA, PFMOPrA or PFMOBA for 30 days, Table S5: Mean (±SD) number of thymic lymphocytes, adjusted to the total number of cells in the thymus (cellularity) of female C57BL/6 mice (*n* = 6) orally exposed to PFMOAA, PFMOPrA or PFMOBA for 30 days, Table S6: Mean (±SD) number of splenic lymphocytes, adjusted to the total number of cells in the thymus (cellularity) of male C57BL/6 mice (*n* = 6) orally exposed to PFMOAA, PFMOPrA or PFMOBA for 30 days, Table S7: Mean (±SD) number of splenic lymphocytes, adjusted to the total number of cells in the thymus (cellularity) of female C57BL/6 mice (*n* = 6) orally exposed to PFMOAA, PFMOPrA or PFMOBA for 30 days, Table S8: Mean (±SD) absolute number of splenic B and natural killer cells from male and female C57BL/6 mice orally exposed to PFMOAA, PFMOPrA or PFMOBA for 30 days.
