# Correction: Woodlief et al. Immunotoxicity of Per- and Polyfluoroalkyl Substances: Insights into Short-Chain PFAS Exposure. *Toxics* 2021, *9*, 100

**DOI:** 10.3390/toxics11080656

**Published:** 2023-07-28

**Authors:** Tracey Woodlief, Samuel Vance, Qing Hu, Jamie DeWitt

**Affiliations:** Department of Pharmacology and Toxicology, Brody School of Medicine, East Carolina University, Greenville, NC 27858, USAhuq@ecu.edu (Q.H.); dewittj@ecu.edu (J.D.)

## Error in Figure/Table

Error in figure x-axis (Figure 1A: Hepatic peroxisome proliferation). In the original publication [1] there was a mistake in Figure 1A as published. The x-axis was supposed to be labeled with the following doses: 0, 0.00025, 0.025, or 2.5 PFOA. The authors state that the scientific conclusions are unaffected. This correction was approved by the Academic Editor. The original publication has also been updated.

## Figures and Tables

**Figure 1:**
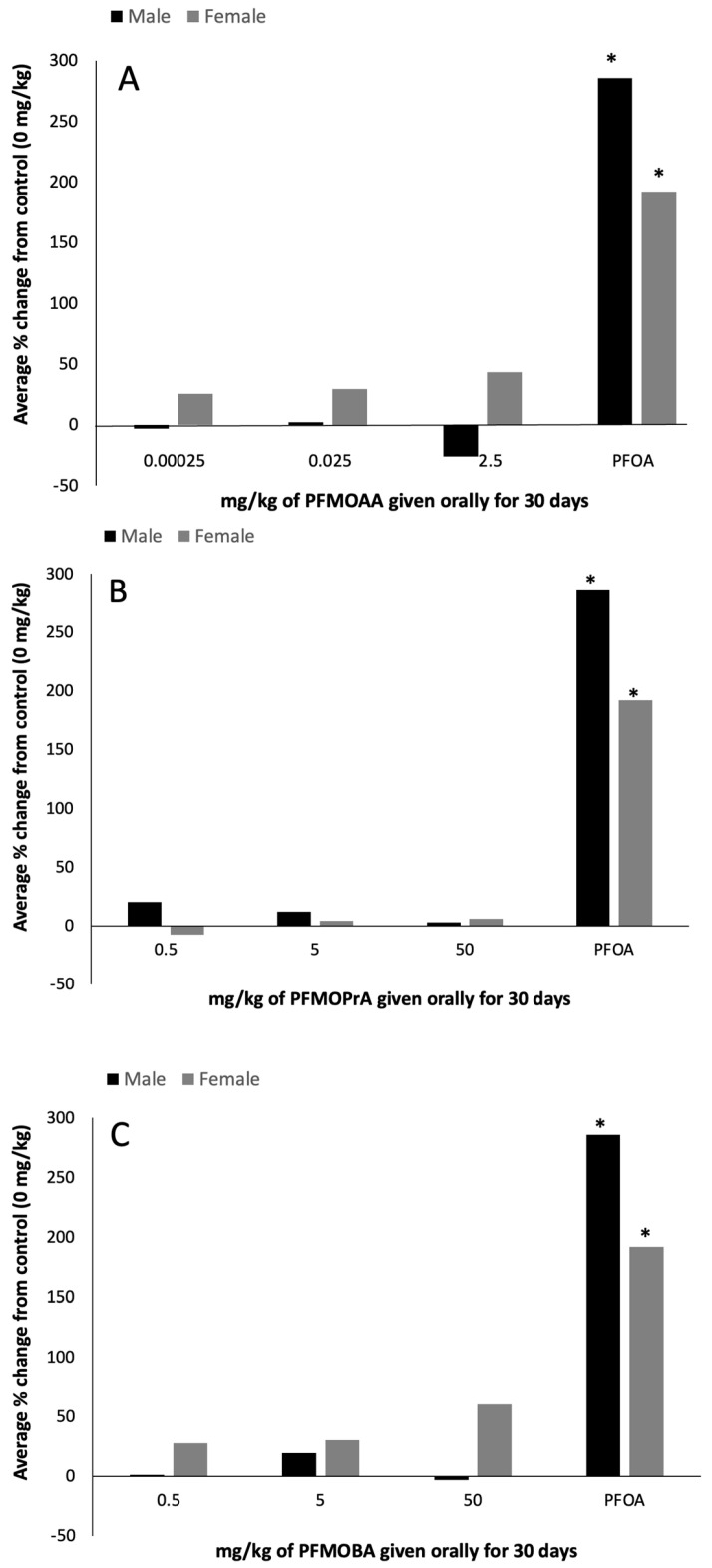
Hepatic peroxisome proliferation (percent change from 0 mg/kg control) of male and female C57BL/6 mice orally exposed to (**A**): PFMOAA, (**B**): PFMOPrA, or (**C**): PFMOBA for 30 days. Acyl-CoA oxidase activity was measured in livers that had been collected from animals one day after exposure ended. *n* = 4–6/dose for PFMOAA, PFMOPrA, PFMOBA, and PFOA-positive control (note that the PFOA-positive control was included from animals evaluated in a separate PFAS study). No error bars are present due to how the data were calculated. Abbreviations: perfluoro-2-methoxyacetic acid (PFMOAA), perfluoro-2-methoxypropanoic acid (PFMOPrA), perfluoro-4-methoxybutanioc acid (PFMOBA), and perfluorooctanoic acid (PFOA). * *p* < 0.05 from same-sex control group.
