# Supplementary material for: Immunotoxicity of Per- and Polyfluoroalkyl Substances: Insights into Short-Chain PFAS Exposure
Source: Toxics. 2021 May 1;9(5):100. doi: 10.3390/toxics9050100 (PMC8147192; doi:10.3390/toxics9050100)
Supplement: Supplementary file 1 [file toxics-09-00100-s001.zip › toxics-1169087-supplementary.pdf]

Supplementary Materials: Immunotoxicity of Per- and Polyfluoroalkyl Substances: In-sights into Short-Chain PFAS Exposure

Tracey Woodlief *, Samuel Vance, Qing Hu and Jamie DeWitt

Table S1. Mean (± standard deviation) serum PFMOAA, PFMOPrA or PFMOBA concentrations in male and female C57BL/6 mice (*n*= 4–6 per group) orally exposed to PFMOAA, PFMOPrA or PFMOBA for 30-days. Samples were collected one day after dosing ended and were pooled per cage for analysis (*n* = 3 mice/tube; *n* = 2 tubes per dose group).

**Male**		**Female**	
Dose group (mg/kg)	PFMOAA (ng/mL)	Dose group (mg/kg)	PFMOAA (ng/mL)
0.0	< LOD (0.5) U	0	< LOD (0.5) U
0.00025	< LOD (0.5) U	0.00025	< LOD (0.5) U
0.025	< LOD (0.5) U	0.025	< LOD (0.5) U
2.5	< LOD (0.5) U	2.5	< LOD (0.5) U
Dose group (mg/kg)	PFMOPrA (ng/mL)	Dose group (mg/kg)	PFMOPrA (ng/mL)
0.0	< LOD (1.2) U	0.0	< LOD (1.2) U
0.5	48.00 ± 9.33	0.5	< LOD (1.2) U
5.0	366.50 ± 142.13	5.0	4.13 ± 0.59
50	872.50 ± 392.44	50	28.40 ± 6.08
Dose group (mg/kg)	PFMOBA (ng/mL)	Dose group (mg/kg)	PFMOBA (ng/mL)
0.0	< LOD (7.4) U	0.0	< LOD (7.4) U
0.5	< LOD (7.4) U	0.5	< LOD (7.4) U
5.0	< LOD (7.4) U	5.0	< LOD (7.4) U
50	< LOD (7.4) U	50	< LOD (7.4) U

Abbreviations: perfluoro-2-methoxyacetic acid (PFMOAA), perfluoro-2-methoxypropanoic acid (PFMOPrA), perfluoro-4-methoxybutanioc acid (PFMOBA) Level of detection (LOD), Units (U).

Table S2. Mean (± standard deviation) body and relative organ weights of male C57BL/6 mice (*n* = 4–6 per group) orally exposed to PFMOAA, PFMOPrA, or PFMOBA for 30 days. Weights were collected one day after dosing ended.

**PFMOAA**	**Group 1 (0.0 mg/kg)**	**Group 2 (0.00025 mg/kg)**	**Group 3 (0.025 mg/kg)**	**Group 4 (2.5 mg/kg)**
Terminal body weight (g)	23.73 ± 1.28	24.42 ± 2.46	24.45 ± 1.50	24.30 ± 1.36
Terminal liver-free body weight (g)	22.54 ± 1.20	23.22 ± 2.32	23.16 ± 1.41	23.03 ± 1.29
Spleen (mg/g)	3.36 ± 0.33	3.45 ± 1.18	3.82 ± 0.75	3.91 ± 1.24
Thymus (mg/g)	2.29 ± 0.39	2.00 ± 0.26	2.19 ± 0.34	2.49 ± 0.44
Brain (mg/g)	16.97 ± 1.03	16.77 ± 1.14	16.42 ± 0.91	16.47 ± 0.61
Liver (mg/g)	50.39 ± 1.21	48.76 ± 2.88	52.64 ± 1.84	52.19 ± 4.25
Heart (mg/g)	4.79 ± 0.43	4.79 ± 0.45	4.96 ± 0.60	5.15 ± 0.53
Kidney (mg/g)	12.22 ± 0.40	11.92 ± 0.67	12.36 ± 0.60	12.60 ± 0.67
PFMOPrA	Group 1 (0.0 mg/kg)	Group 2 (0.5 mg/kg)	Group 3 (5.0 mg/kg)	Group 4 (50 mg/kg)
Terminal body weight (g)	24.10 ± 1.15	25.58 ± 1.21	25.36 ± 1.36	25.82 ± 1.15
Terminal liver-free body weight (g)	22.89 ± 1.10	24.28 ± 1.17	24.04 ± 1.25	24.15 ± 1.02
Spleen (mg/g)	3.92 ± 0.70	3.54 ± 0.45	3.37 ± 0.26	3.48 ± 0.36

Thymus (mg/g)	2.76 ± 0.74	2.57 ± 0.53	2.45 ± 0.19	2.09 ± 0.96
Brain (mg/g)	17.45 ± 0.73	15.87 ± 0.74	16.16 ± 0.61	16.08 ± 0.71
Liver (mg/g)	50.20 ± 1.49	50.52 ± 1.15	52.03 ± 2.79	64.50 ± 6.47
Heart (mg/g)	4.67 ± 0.27	5.43 ± 0.54	5.11 ± 0.70	5.00 ± 0.37
Lungs (mg/g)	6.69 ± 0.97	9.21 ± 2.17	8.67 ± 2.40	7.39 ± 0.63
Kidneys (mg/g)	12.75 ± 1.04	13.00 ± 1.16	14.03 ± 1.04	13.37 ± 0.71
PFMOBA	Group 1 (0.0 mg/kg)	Group 2 (0.5 mg/kg)	Group 3 (5.0 mg/kg)	Group 4 (50 mg/kg)
Terminal body weight (g)	24.92 ± 2.29	26.52 ± 1.91	26.10 ± 2.51	24.53 ± 2.19
Terminal liver-free body weight (g)	23.76 ± 2.23	25.19 ± 1.79	24.81 ± 2.36	23.21 ± 2.02
Spleen (mg/g)	4.10 ± 1.51	4.19 ± 1.11	3.81 ± 1.23	4.89 ± 1.09
Thymus (mg/g)	2.50 ± 0.75	2.07 ± 0.42	2.13 ± 0.58	2.21 ± 0.28
Brain (mg/g)	16.68 ± 1.57	15.38 ± 0.70	15.59 ± 0.77	16.58 ± 1.63
Liver (mg/g)	46.73 ± 5.34	49.87 ± 2.27	49.51 ± 2.15	53.61 ± 3.68*
Heart (mg/g)	4.86 ± 0.83	5.11 ± 0.30	5.62 ± 0.82	5.18 ± 0.96
Lungs (mg/g)	7.28 ± 1.69	6.50 ± 0.79	6.92 ± 0.42	7.09 ± 1.99
Kidney (mg/g)	12.62 ± 1.50	12.74 ± 0.76	13.09 ± 0.56	12.55 ± 1.10
PFOA	7.5 mg/kg			
Terminal body weight (g)	21.8 ± 1.14			
Terminal liver-free body weight (g)	19.05 ± 0.96*			
Spleen (mg/kg)	3.38 ± 0.44			
Thymus (mg/kg)	2.48 ± 0.28			
Liver (mg/kg)	125.4 ± 9.91*			

Abbreviations: perfluoro-2-methoxyacetic acid (PFMOAA), perfluoro-2-methoxypropanoic acid (PFMOPrA), perfluoro-4-methoxybutanioc acid (PFMOBA), mg organ weight/ body weight (mg/g) grams (g), and milligrams (mg). *$p < 0.05$ versus control.

Table S3. Mean (± standard deviation) body and relative organ weights of female C57BL/6 mice (n = 4–6 per group) orally exposed to PFMOAA, PFMOPrA, or PFMOBA for 30 days. Weights were collected one day after dosing ended.

**PFMOAA**	**Group 1 (0.0 mg/kg)**	**Group 2 (0.00025 mg/kg)**	**Group 3 (0.025 mg/kg)**	**Group 4 (2.5 mg/kg)**
Terminal body weight (g)	21.20 ± 1.06	21.87 ± 1.07	21.45 ± 0.92	20.66 ± 2.12
Terminal Liver free body weight (g)	20.23 ± 1.00	20.81 ± 1.04	20.41 ± 0.86	19.65 ± 1.96
Spleen (mg/g)	4.35 ± 0.41	4.79 ± 0.89	4.88 ± 0.27	4.51 ± 0.43
Thymus (mg/g)	3.48 ± 0.44	3.30 ± 0.54	3.14 ± 0.36	3.41 ± 0.79
Brain (mg/g)	19.48 ± 1.29	19.23 ±0.74	19.09 ± 0.82	20.28 ± 1.69
Liver (mg/g)	45.66 ± 2.69	48.43 ± 3.04	48.33 ± 1.49	48.72 ± 4.17
Heart (mg/g)	4.81 ± 0.63	5.50 ± 0.41	5.29 ± 0.45	5.66 ± 0.80
Kidney (mg/g)	11.95 ± 0.92	12.36 ± 0.77	11.80 ± 0.72	12.35 ± 0.61
PFMOPrA	Group 1 (0.0 mg/kg)	Group 2 (0.5 mg/kg)	Group 3 (5.0 mg/kg)	Group 4 (50 mg/kg)
Terminal body weight (g)	22.60 ± 2.05	22.07 ± 1.72	23.25 ± 0.85	22.07 ± 1.49
Terminal Liver free body weight (g)	21.50 ± 1.91	21.04 ± 1.62	22.13 ± 0.77	21.00 ± 1.38
Spleen (mg/g)	4.92 ± 0.27	4.83 ± 0.33	4.72 ± 0.40	4.59 ± 0.29
Thymus (mg/g)	3.31 ± 0.38	3.30 ± 0.28	3.33 ± 0.42	2.80 ± 0.25
Brain (mg/g)	18.59 ± 1.20	18.85 ± 1.10	17.90 ± 0.71	17.04 ± 1.23
Liver (mg/g)	48.52 ± 3.29	46.35 ± 2.75	48.23 ± 2.90	42.77 ± 2.90
Heart (mg/g)	5.86 ± 0.58	5.46 ± 0.60	4.71 ± 0.36	4.77 ± 0.39
Lungs (mg/g)	7.44 ± 1.03	9.49 ± 2.59	10.04 ± 2.48	8.20 ± 1.74
Kidney (mg/g)	10.46 ± 0.36	11.71 ± 1.24	11.75 ± 0.41	10.69 ± 0.66
PFMOBA	Group 1 (0.0 mg/kg)	Group 2 (0.5 mg/kg)	Group 3 (5.0 mg/kg)	Group 4 (50 mg/kg)
Terminal body weight (g)	20.83 ± 2.07	21.14 ± 1.02	21.03 ± 1.59	20.97 ± 1.43
Terminal Liver free body weight (g)	19.91 ± 1.98	20.12 ± 0.95	20.02 ± 1.56	19.97 ± 1.37
Spleen (mg/g)	5.03 ± 0.54	4.91 ± 0.58	6.17 ± 3.47	4.77 ± 0.76
Thymus (mg/g)	3.44 ± 0.51	3.33 ± 0.59	3.68 ± 0.90	3.56 ± 0.55

Brain (mg/g)	20.45 ± 1.41	19.49 ± 0.64	19.41 ± 1.21	19.32 ± 1.24
Liver (mg/g)	44.10 ± 6.48	48.21 ± 2.99	48.45 ± 2.87	47.62 ± 2.64
Heart (mg/g)	5.15 ± 0.68	5.13 ± 0.64	5.75 ± 1.06	5.19 ± 0.76
Lungs (mg/g)	8.32 ± 0.84	8.23 ± 0.95	9.03 ± 1.23	8.40 ± 2.08
Kidney (mg/g)	10.98 ± 0.90	11.10 ± 0.44	11.30 ± 1.14	10.93 ± 1.94
PFOA	7.5 mg/kg			
Terminal body weight (g)	17.2 ± 0.88*			
Terminal liver-free body weight (g)	14.49 ± 0.68*			
Spleen (mg/kg)	2.79 ± 0.38*			
Thymus (mg/kg)	2.97 ± 1.58			
Liver (mg/kg)	156.17 ± 4.66*			

Abbreviations: perfluoro-2-methoxyacetic acid (PFMOAA), perfluoro-2-methoxypropanoic acid (PFMOPrA), perfluoro-4-methoxybutanioc acid (PFMOBA), mg organ weight/ body weight (mg/g), grams (g), and milligrams (mg). *$p < 0.05$ versus control.

Table S4. Mean (± standard deviation) number of thymic lymphocytes, adjusted to the total number of cells in the thymus (cellularity) of male C57BL/6 mice (n = 4–6 per group) orally exposed to PFMOAA, PFMOPrA, or PFMOBA for 30 days. Cells were analyzed by flow cytometry one day after dosing ended.

**PFMOAA**	**Organ total cellularity (e^7)**	**Cells/mg organ weight (e^6)**	**CD4$^+$(e^5)**	**CD8$^+$(e^6)**	**CD4$^+$ CD8$^+$ (e^6)**	**CD4$^-$ CD8$^-$ (e^5)**
0.0 mg/kg	6.35 ± 1.42	1.19 ± 0.29	6.78 ± 1.66	2.69 ± 0.42	2.27 ± 0.55	3.25 ± 0.93
0.00025 mg/kg	7.66 ± 1.31	1.58 ± 0.18	10.82 ± 2.67	4.24 ± 0.97	3.03 ± 0.69	4.80 ± 1.41
0.025 mg/kg	7.73 ± 2.11	1.45 ± 0.35	11.57 ± 2.82	4.31 ± 0.93	3.23 ± 0.96	4.42 ± 1.71
2.5 mg/kg	7.47 ± 1.17	1.27 ± 0.29	8.00 ± 1.57	3.35 ± 0.99	2.81 ± 0.47	4.15 ± 0.98
PFMOPrA	Organ total cellularity (e^7)	Cells/mg organ weight (e^6)	CD4$^+$(e^5)	CD8$^+$(e^5)	CD4$^+$ CD8$^+$ (e^5)	CD4$^-$ CD8$^-$ (e^4)
0.0 mg/kg	8.98 ± 1.07	1.36 ± 0.30	5.87 ± 1.63	1.29 ± 0.31	4.88 ± 0.61	7.03 ± 2.57
0.5 mg/kg	9.08 ± 0.98	1.44 ± 0.37	6.32 ± 1.71	1.43 ± 0.41	5.19 ± 1.48	5.65 ± 1.45
5.0 mg/kg	8.26 ± 1.10	1.33 ± 0.15	5.59 ± 0.65	1.26 ± 0.20	5.03 ± 0.63	6.13 ± 1.95
50 mg/kg	8.89 ± 0.66	1.56 ± 0.35	6.28 ± 1.32	1.63 ± 0.55	5.98 ± 1.39	7.88 ± 2.98
PFMOBA	Organ total cellularity (e^7)	Cells/mg organ weight (e^5)	CD4$^+$(e^5)	CD8$^+$(e^4)	CD4$^+$ CD8$^+$ (e^4)	CD4$^-$ CD8$^-$ (e^4)
0.0 mg/kg	3.40 ± 1.56	5.57 ± 2.81	3.03 ± 1.63	5.74 ± 3.97	7.65 ± 4.34	3.51 ± 1.75
0.5 mg/kg	3.73 ± 0.91	7.08 ± 2.40	3.98 ± 1.30	7.88 ± 3.19	11.78 ± 6.83	4.03 ± 1.27
5.0 mg/kg	4.13 ± 0.85	7.98 ± 3.01	4.59 ± 1.73	10.13 ± 4.76	9.83 ± 4.08	5.40 ± 1.82
50 mg/kg	3.89 ± 0.21	7.30 ± 0.10	4.06 ± 0.68	9.47 ± 1.61	110.54 ± 246.14	3.89 ± 0.67

Data are presented as the mean cell number ± standard deviation based on a total of 10,000 events. n = 4–6 animals/sex/dose. Abbreviations: perfluoro-2-methoxyacetic acid (PFMOAA), perfluoro-2-methoxypropanoic acid (PFMOPrA), and perfluoro-4-methoxybutanioc acid (PFMOBA).

Table S5. Mean (± standard deviation) absolute number of thymic lymphocytes, adjusted to the total number of cells in the thymus (cellularity) of female C57BL/6 mice (n = 4–6 per group) orally exposed to PFMOAA, PFMOPrA, or PFMOBA for 30 days. Cells were analyzed by flow cytometry one day after dosing ended.

**PFMOAA_**	**Organ total cellularity (e^7)**	**Cells/mg organ weight (e^5)**	**CD4$^+$(e^6)**	**CD8$^+$(e^6)**	**CD4$^+$ CD8$^+$ (e^6)**	**CD4$^-$ CD8$^-$ (e^5)**
0.0 mg/kg	7.21 ± 1.91	1.75 ± 0.45	1.02 ± 0.25	4.05 ± 0.32	1.82 ± 0.70	4.92 ± 1.57
0.00025 mg/kg	5.03 ± 1.60	1.19 ± 0.35	0.57 ± 0.22	2.75 ± 0.79	0.95 ± 0.42	5.12 ± 2.46
0.025 mg/kg	6.02 ± 2.82	1.47 ± 0.67	0.57 ± 0.29	2.87 ± 1.44	1.07 ± 0.71	5.23 ± 1.94
2.5 mg/kg	8.45 ± 1.76	2.03 ± 0.41	1.13 ± 0.28	4.48 ± 1.02	1.64 ± 0.55	3.99 ± 1.09
PFMOPrA_	Organ total cellularity (e^7)	Cells/mg organ weight (e^6)	CD4$^+$(e^7)	CD8$^+$(e^6)	CD4$^+$ CD8$^+$ (e^7)	CD4$^-$ CD8$^-$ (e^6)
0.0 mg/kg	8.17 ± 1.33	1.10 ± 0.19	3.27 ± 0.24	7.06 ± 1.42	2.92 ± 3.39	3.93 ± 1.20
0.5 mg/kg	8.36 ± 3.89	1.15 ± 0.53	2.83 ± 0.29	7.47 ± 1.07	2.55 ± 0.61	3.91 ± 0.60
5.0 mg/kg	9.65 ± 3.85	1.26 ± 0.50	3.76 ± 0.39	9.45 ± 0.98	3.04 ± 1.42	5.07 ± 0.61
50 mg/kg	10.84 ± 1.67	1.56 ± 0.24	2.31 ± 0.33	5.76 ± 1.00	2.51 ± 0.33	2.88 ± 0.58
PFMOBA	Organ total cellularity (e^7)	Cells/mg organ weight (e^5)	CD4$^+$(e^5)	CD8$^+$(e^4)	CD4$^+$ CD8$^+$ (e^5)	CD4$^-$ CD8$^-$ (e^4)
0.0 mg/kg	4.31 ± 1.13	6.28 ± 2.33	2.70 ± 0.99	5.56 ± 1.72	1.86 ± 0.63	3.30 ± 1.32

0.5 mg/kg	4.87 ± 1.06	7.02 ± 1.45	3.12 ± 0.70	5.42 ± 1.91	2.17 ± 0.70	4.24 ± 1.24
5.0 mg/kg	6.56 ± 1.45	8.75 ± 2.30	2.59 ± 1.00	7.26 ± 1.71	2.59 ± 1.00	5.03 ± 0.21
50 mg/kg	5.01 ± 0.74	6.87 ± 1.34	1.68 ± 0.82	5.84 ± 1.58	1.68 ± 0.82	4.55 ± 0.10

Data are presented as the mean cell number ± standard deviation based on a total of 10,000 events. *n* = 4–6 per group. Abbreviations: perfluoro-2-methoxyacetic acid (PFMOAA), perfluoro-2-methoxypropanoic acid (PFMOPrA), and perfluoro-4-methoxybutanioc acid (PFMOBA).

Table S6. Mean (± standard deviation) absolute number of splenic lymphocytes, adjusted to the total number of cells in the spleen (cellularity) of male C57BL/6 mice (*n* = 4–6 per group) orally exposed to PFMOAA, PFMOPrA, or PFMOBA for 30 days. Cells were analyzed by flow cytometry one day after dosing ended.

**PFMOAA**	**Organ total cellularity (e⁷)**	**Cells/mg organ weight (e⁵)**	**CD4⁺(e⁶)**	**CD8⁺(e⁶)**	**CD4⁺ CD8⁺ (e⁴)**	**CD4⁻ CD8⁻ (e⁵)**
0.0 mg/kg	3.29 ± 1.31	4.22 ± 1.81	3.91 ± 1.52	6.46 ± 3.12	1.30 ± 0.78	6.41 ± 2.25
0.00025 mg/kg	2.83 ± 1.17	3.59 ± 1.79	3.79 ± 1.39	5.49 ± 2.70	1.59 ± 1.10	6.62 ± 1.91
0.025 mg/kg	4.42 ± 2.23	5.03 ± 3.14	4.95 ± 2.46	8.48 ± 5.07	1.02 ± 0.34	8.50 ± 3.88
2.5 mg/kg	6.01 ± 1.94	6.34 ± 0.81	6.23 ± 1.21	12.55 ± 3.47	3.73 ± 2.30	12.10 ± 0.42
PFMOPrA	Organ total cellularity (e⁷)	Cells/mg organ weight (e⁵)	CD4⁺(e⁵)	CD8⁺(e⁴)	CD4⁺ CD8⁺ (e²)	CD4⁻ CD8⁻ (e³)
0.0 mg/kg	5.60 ± 0.18	6.12 ± 1.26	1.29 ± 0.17	7.27 ± 1.30	3.68 ± 1.20	8.13 ± 1.85
0.5 mg/kg	5.81 ± 1.09	6.39 ± 0.56	1.20 ± 0.13	6.43 ± 1.05	2.23 ± 1.98	7.46 ± 1.29
5.0 mg/kg	3.75 ± 0.74	4.39 ± 0.80	0.90 ± 0.17	5.11 ± 0.91*	2.50 ± 0.82	5.31 ± 1.15
50 mg/kg	5.95 ± 1.39	6.66 ± 1.65	1.26 ± 0.31	6.91 ± 1.59	2.67 ± 1.81	7.51 ± 1.56
PFMOBA	Organ total cellularity (e⁷)	Cells/mg organ weight (e⁵)	CD4⁺(e⁴)	CD8⁺(e⁴)	CD4⁺ CD8⁺ (e²)	CD4⁻ CD8⁻ (e³)
0.0 mg/kg	2.29 ± 1.02	2.24 ± 0.99	4.27 ± 2.21	2.21 ± 1.10	1.16 ± 0.79	2.93 ± 0.86
0.5 mg/kg	4.44 ± 0.57	4.16 ±0.73	7.64 ± 1.15	3.98 ± 0.71	1.78 ± 0.66	4.43 ± 0.30
5.0 mg/kg	4.21 ± 0.77	4.57 ± 1.45	8.20 ± 2.67	4.18 ± 1.59	1.70 ± 0.65	4.96 ± 1.60
50 mg/kg	4.08 ± 0.64	3.52 ± 0.71	5.62 ± 2.32	2.82 ± 1.16	1.34 ± 0.86	4.05 ± 0.53*

Data are presented as the mean cell number ± standard deviation based on a total of 10,000 events. N= 4-6per group. Abbreviations: perfluoro-2-methoxyacetic acid (PFMOAA), perfluoro-2-methoxypropanoic acid (PFMOPrA), and perfluoro-4-methoxybutanioc acid (PFMOBA) * $p < 0.05$ versus control.

Table S7. Mean (± standard deviation) absolute number of splenic lymphocytes, adjusted to the total number of cells in the spleen (cellularity) of female C57BL/6 mice (*n* = 4–6 per group) orally exposed to PFMOAA, PFMOPrA, or PFMOBA for 30 days. Cells were analyzed by flow cytometry one day after dosing ended.

**PFMOAA_**	**Organ total cellularity (e⁷)**	**Cells/mg organ weight (e⁵)**	**CD4⁺(e⁶)**	**CD8⁺(e⁶)**	**CD4⁺ CD8⁺ (e⁴)**	**CD4⁻ CD8⁻ (e⁵)**
0.0 mg/kg	4.51 ± 0.34	5.74 ± 0.48	5.61 ± 0.47	9.76 ± 0.72	2.69 ± 2.40	8.21 ± 1.11
0.00025 mg/kg	4.83 ± 1.90	6.73 ± 2.31	4.94 ± 1.93	8.71 ± 4.22	2.72 ± 1.34	9.13 ± 3.12
0.025 mg/kg	3.21 ± .051	4.80 ± 0.84	3.82 ± 0.67	4.95 ± 0.88	2.09 ± 1.69	6.64 ± 0.83
2.5 mg/kg	4.16 ± 1.71	6.49 ±3.84	4.92 ± 1.80	8.15 ± 3.49	2.78 ± 0.89	7.73 ± 2.19
PFMOPrA_	Organ total cellularity (e⁷)	Cells/mg organ weight (e⁵)	CD4⁺(e⁴)	CD8⁺(e⁴)	CD4⁺ CD8⁺ (e²)	CD4⁻ CD8⁻ (e³)
0.0 mg/kg	7.85 ± 0.76	7.13 ± 0.94	1.48 ± 0.20	7.81 ± 0.51	2.70 ± 1.81	1.07 ± 0.21
0.5 mg/kg	6.97 ± 0.94*	6.55 ± 0.52	1.33 ± 0.01	6.65 ± 0.85	3.46 ± 1.67	1.00 ± 0.12
5.0 mg/kg	9.37 ± .043*	8.60 ± 0.68*	1.70 ± 0.20	8.75 ± 0.50	5.33 ± 2.25	1.31 ± 0.14
50 mg/kg	6.04 ± 0.53*	6.02 ± *0.83	1.25 ± 0.01	6.91 ± 0.54	4.07 ± 1.21	0.94 ± 0.16
PFMOBA	Organ total cellularity (e⁷)	Cells/mg organ weight (e⁵)	CD4⁺(e⁵)	CD8⁺(e⁴)	CD4⁺ CD8⁺ (e²)	CD4⁻ CD8⁻ (e⁴)
0.0 mg/kg	8.49 ± 2.63	8.38 ± 3.07	1.65 ± 0.58	9.25 ± 0.35	7.73 ± 4.15	1.65 ± 0.66
0.5 mg/kg	7.01 ± 2.87	6.63 ± 2.35	1.41 ± 0.55	7.73 ± 0.27	5.99 ± 3.56	1.15 ± 0.35
5.0 mg/kg	8.36 ± 2.32	7.32 ± 2.85	1.44 ± 0.66	7.47 ± 0.36	6.85 ± 3.18	1.25 ± 0.49
50 mg/kg	4.82 ± 1.66	5.20 ± 3.06	1.13 ± 0.70	6.20 ± 0.54	6.07 ± 3.68	0.91 ± 0.50

Data are presented as the mean cell number ± standard deviation based on a total of 10,000 events. *n* = 4-6 per group. Abbreviations: perfluoro-2-methoxyacetic acid (PFMOAA), perfluoro-2-methoxypropanoic acid (PFMOPrA), and perfluoro-4-methoxybutanioc acid (PFMOBA) * $p < 0.05$ versus control.

Table S8. Mean (± standard deviation) absolute number of splenic B and natural killer cells from male and female C57BL/6 mice orally exposed to PFMOAA, PFMOPrA, or PFMOBA for 30 days. Cells were analyzed by flow cytometry one day after dosing ended.

PFMOAA *Male*	B cell (e^7)	NK cell (e^6)	PFMOAA *Female*	B cell (e^7)	NK cell (e^6)
0.0 mg/kg	1.48 ± 0.71	1.01 ± 0.37	0.0 mg/kg	2.24 ± 0.02	1.37 ± 0.07
0.00025 mg/kg	1.22 ± 0.69	1.11 ± 0.35	0.00025 mg/kg	2.17 ± 1.00	1.35 ± 0.42
0.025 mg/kg	2.33 ± 1.30	1.22 ± 0.41	0.025 mg/kg	1.26 ± 0.20	1.01 ± 0.23
2.5 mg/kg	2.93 ± 0.75	1.85 ± 0.56	2.5 mg/kg	1.86 ± 0.88	0.89 ± 0.14
PFMOPrA *Male*	B cell (e^7)	NK cell (e^6)	PFMOPrA *Female*	B cell (e^7)	NK cell (e^6)
0.0 mg/kg	1.80 ± 0.14	1.45 ± 0.28	0.0 mg/kg	1.80 ± 0.15	1.98 ± 0.36
0.5 mg/kg	2.14 ± 0.25	1.54 ± 0.22	0.00025 mg/kg	1.55 ± 0.25	1.86 ± 0.28
5.0 mg/kg	1.12 ± 0.21	0.81 ± 0.23	0.025 mg/kg	2.23 ± 0.20	2.27 ± 0.36
50 mg/kg	2.06 ± 0.50	1.35 ± 0.24	2.5 mg/kg	1.19 ± 0.38	1.56 ± 0.45
PFMOBA *Male*	B cell (e^6)	NK cell (e^5)	PFMOBA *Female*	B cell (e^7)	NK cell (e^6)
0.0 mg/kg	5.50 ± 2.46	5.06 ± 2.16	0.0 mg/kg	1.92 ± 0.62	2.27 ± 0.85
0.5 mg/kg	10.90 ± 1.20*	10.42 ± 1.61*	0.00025 mg/kg	1.48 ± 0.59	1.73 ± 0.75
5.0 mg/kg	11.07 ± 2.16*	9.95 ± 2.04*	0.025 mg/kg	1.64 ± 0.39	1.82 ± 0.51
50 mg/kg	8.83 ± 1.72*	9.16 ± 2.11*	2.5 mg/kg	1.00 ± 0.31*	1.04 ± 0.45*

Data are presented as the mean cell number ± standard deviation based on a total of 10,000 events. n = 4–6 animals per group.* Abbreviations: perfluoro-2-methoxyacetic acid (PFMOAA), perfluoro-2-methoxypropanoic acid (PFMOPrA), perfluoro-4-methoxybutanioc acid (PFMOBA), and natural killer (NK). * $p < 0.05$ versus control.
